# A Combination of Factors Related to Smoking Behavior, Attractive Product Characteristics, and Socio-Cognitive Factors are Important to Distinguish a Dual User from an Exclusive E-Cigarette User

**DOI:** 10.3390/ijerph16214191

**Published:** 2019-10-30

**Authors:** Kim A. G. J. Romijnders, Jeroen L. A. Pennings, Liesbeth van Osch, Hein de Vries, Reinskje Talhout

**Affiliations:** 1 Centre for Health Protection, National Institute for Public Health and the Environment (RIVM), Antonie van Leeuwenhoeklaan, 9, 3721 MA Bilthoven, The NetherlandsReinskje.talhout@rivm.nl (R.T.); 2 Department of Health Promotion, CAPHRI School for Public Health and Primary Care, Maastricht University, POB 616 6200 MD Maastricht, The Netherlands; Liesbeth.vanosch@maastrichtuniversity.nl (L.v.O.); hein.devries@maastrichtuniversity.nl (H.d.V.)

**Keywords:** e-cigarettes, dual use, public health, machine learning, random forest, smoking behavior, attractiveness, socio-cognitive factors

## Abstract

Although total cessation of nicotine and tobacco products would be most beneficial to improve public health, exclusive e-cigarette use has potential health benefits for smokers compared to cigarette smoking. This study investigated differences between dual users and exclusive e-cigarette users provide information to optimize health communication about smoking and vaping. A cross-sectional survey (n = 116) among 80 current, adult dual users and 36 current, adult-exclusive e-cigarette users was conducted in the Netherlands. The questionnaire assessed four clusters of factors: (1) Past and current smoking and vaping behavior, (2) product characteristics used, (3) attractiveness and reasons related to cigarettes and e-cigarettes, and (4) socio-cognitive factors regarding smoking, vaping, and not smoking or vaping. We used random forest—a machine learning algorithm—to identify distinguishing features between dual users and e-cigarette users. We are able to discern a dual user from an exclusive e-cigarette user with 86.2% accuracy based on seven factors: Social ties with other smokers, quantity of tobacco cigarettes smoked in the past (e-cigarette users) or currently (dual users), self-efficacy to not vape and smoke, unattractiveness of cigarettes, attitude towards e-cigarettes, barriers: accessibility of e-cigarettes, and intention to quit vaping (A). This combination of features provides information on how to improve health communication about smoking and vaping.

## 1. Introduction

The use of electronic cigarettes (e-cigarettes) has increased worldwide in recent years [1,2]. The majority of the e-cigarette users are currently former cigarette smokers that either switched completely to e-cigarettes or use e-cigarettes in addition to tobacco cigarettes [2,3,4,5,6,7]. Concerns exist that e-cigarette use may attract (adolescent) never users, and thus, affect public health adversely, or even lead to dual use and a nicotine addiction [8,9,10]. Although never use or total cessation of all nicotine and tobacco products would be most beneficial for all [8,11,12], exclusive e-cigarette use—and not dual use of both cigarettes and e-cigarettes, which has an adverse public health effect [6,8,13]—has potential health benefits for smokers compared to cigarette smoking [13,14,15,16,17,18]. However, exclusive e-cigarette use, especially among never-users, is not without risks itself [8]. Consequently, it is recommended to be prevented among non-smokers [8]. To improve public health, it is important to optimize health communication about smoking and vaping to the needs of never users, non-current users, exclusive smokers, dual users, and exclusive e-cigarette users. Since dual users and exclusive e-cigarette users are distinct groups [13], their information and communication needs are likely different. The present paper aims to increase our understanding of these two distinct groups and provide insight into factors that can be targeted with health communication.

Research has presented several differences between exclusive e-cigarette users and dual users, but the variability in results and definitions used makes it difficult to compare these differences [19]. Review of relevant literature has identified four clusters of differences between dual users and e-cigarette users [9,13,19,20,21,22,23,24,25,26,27,28,29,30,31,32,33,34]. While previous research looked at differences between dual users and exclusive e-cigarette users on an individual factor level or combined two clusters in an analysis, the goal of our study is to provide aggregated data by combining all the clusters of factors identified with machine learning [9,13,19,20,21,22,23,24,25,26,27,28,29,30,31,32,33,34]. First, differences were found in current smoking behavior among dual users or past smoking behavior among e-cigarette users, and current vaping behavior. Research has found that dual users smoked for a longer time (i.e., more pack years) compared to e-cigarette users [9,21]. Additionally, the number of cigarettes smoked per day increased the likelihood of initiating e-cigarette use [9,22], but no differences were found between dual users and e-cigarette users [23]. Smith, Gawron, Balwicki, Sobczak, Matynia and Goniewicz [9] have demonstrated that experimenting with e-cigarette use increased over time from 20% in 2011 to 70% in 2016 in Poland, and while rates of exclusive daily smoking declined, rates of exclusive daily vaping increased.

Second, there is variability in differences reported in product characteristics of e-cigarettes used by dual users versus exclusive e-cigarette users. One study reported that dual users were more likely than exclusive e-cigarette users to use nicotine-containing e-liquids [9]. In comparison, other studies found that nicotine concentration in e-liquids used did not differ between dual users and exclusive e-cigarette users [23,24]. Similarly, this variability in reported differences is observed in e-liquid flavors used. While one study found no differences between dual users and e-cigarette users in flavors used [9], another study found that tobacco e-liquid flavors were more popular among dual users than e-cigarette users [25]. These differences in product characteristics of e-cigarettes used may partly be explained by differences in regulations across countries. In the Netherlands, product characteristics of e-cigarettes, such as maximum levels of nicotine per e-liquid (20mg/mL in the Netherlands), and the maximum volume of e-liquids (10mL in the Netherlands) are regulated through the Tobacco regulation [35,36].

Third, differences have been observed in the perceived attractiveness of product characteristics of cigarettes and e-cigarettes [19,25,26,27,28]. Dual users and exclusive e-cigarette users differed in how they perceived the attractiveness of the ability to avoid smoking restrictions with e-cigarettes [29], lower costs of e-cigarettes compared to cigarettes [24], and the variety of e-liquid flavors available [19,26]. These attractive aspects are a reason to initiate e-cigarette use and may influence a person’s attitude towards e-cigarettes [32,33]. Although attractiveness and reasons related to e-cigarette use have previously been reported to differ between dual users and exclusive e-cigarette users [19,21,23]. Fourth, socio-cognitive determinants regarding e-cigarette use differed between dual users and exclusive e-cigarette users. Dual users were less positive towards e-cigarettes than exclusive e-cigarette users [19,24]. In addition, dual users perceived fewer risks of e-cigarettes compared to smoking than exclusive e-cigarette users [21]. Risk perception differed between dual users and exclusive e-cigarette users [19]. More often, partners, family, friends, and colleagues of e-cigarette users were fellow e-cigarette users [24,30]. E-cigarette users also had less desire for cigarette smoking than dual users, and they found it easier than dual users not to smoke [23,24,30]. Furthermore, while some situations triggered dual users to smoke cigarettes, these situations did not trigger exclusive e-cigarette users [23,24,34]. For example, if dual users experienced stress, were with friends, or had just eaten dinner, they preferred cigarette smoking over vaping [23,24,34]. Dual users were found to be less motivated to quit cigarette smoking or quit nicotine intake altogether than exclusive e-cigarette users [24,25].

While previous research looked at differences between dual users and exclusive e-cigarette users on an individual factor level or combined two clusters in an analysis, the goal of our study is to provide aggregated data [37,38] from the combined four clusters of factors found in previous research with machine learning [9,13,19,20,21,22,23,24,25,26,27,28,29,30,31,32,33,34]. With machine learning, we are able to provide the most relevant features from a variety and a large number of factors identified in the four clusters. These aggregated data could provide information to support the development and improvement of health communication about e-cigarettes in order to prevent the adverse health effects of dual use. With insight into the most relevant features, health communication may be able to target the differences between dual users and exclusive e-cigarette users to aid dual users who would like to switch to exclusive use in the future, or prevent dual use among exclusive users. To explore relevant distinguishing features between adult dual users and adult-exclusive e-cigarette users in a cross-sectional survey, the following four clusters were combined with random forest: (1) Past and current smoking and vaping behavior; (2) product characteristics used; (3) attractiveness and reasons related to cigarettes and e-cigarettes; and (4) socio-cognitive factors regarding smoking, vaping, and not smoking or vaping [9,13,19,20,21,22,23,24,25,26,27,28,29,30,31,32,33,34].

## 2. Materials and Methods 

For the purpose of this study, a cross-sectional survey was conducted among adult (18+), current dual users; and adult (18+), current exclusive e-cigarette users. Adult individuals who currently smoke and use e-cigarettes (i.e., vape) concurrently on a daily or weekly basis, are defined as dual users in the current study [13]. In contrast, adult individuals who exclusively use e-cigarettes on a daily or weekly basis are defined as exclusive e-cigarette users [13]. A full overview of the items reflecting all four clusters of factors used can be found in supplementary file 1. The study was approved by the Medical Ethics Committee of Zuyderland—Zuyd (17-N-88). 

### 2.1. Recruitment

In June 2016, participants were recruited in the Netherlands through an online survey panel (Flycatcher) [39]. This online panel consists of more than 10,000 Dutch individuals and panel participants, who voluntary and actively opt-in (double-active-opt-in). Every year, panelists are asked to update their information. On average panelists complete eight surveys a year. Twelve thousand seven hundred fifty panelists were sent an invitation by email who met the inclusion criteria (being able to understand Dutch, being aware of e-cigarettes, being 13 years or older (adolescent (13–17 years old) and adults (18+))). The questionnaire was administered online, and participants were asked to provide consent before the start of the survey. With a response rate of 10.3%, 1307 participants completed the survey. To distinguish a dual user from an exclusive e-cigarette user, participants were eligible for our current study if they were adults who met the definition of either a dual user or exclusive e-cigarette user. In total, 116 participants (n = 80 adult dual users; n = 36 adult-exclusive e-cigarette users) of this subsample met the definition of dual users and exclusive e-cigarette users. User groups were determined with three questions: Type of user: ‘I smoke or vape…’; frequency of use: ‘How often do you smoke?’; and ‘How often do you vape?’ (see Supplymentary file 1). 

### 2.2. Questionnaire

The current study included measures (see supplementary file 1) on basic demographics and the four identified clusters of differences. The questionnaire was only accessible to Flycatcher panel members with a personalized link. Participants first answered a verification question to make sure that the questionnaire was filled in by the selected participants. All items used were mandatory. Responses were checked on quality by: Time to fill in the questionnaire, consistency in responses, open answer options, and straight lining (e.g., if the same response is chosen in a series of the statement). 

#### 2.2.1. Sociodemographic Measures 

Sociodemographic measures included age, gender, and education. Educational level was determined based on the Dutch version of the International Standard Classification of Education (ISCED) [40] (see supplementary file 1). 

#### 2.2.2. Past and Current Smoking and Vaping Behavior

All participants were asked to report their vaping and smoking behavior. These measures included the type of user, lifetime smoking status [41], frequency of smoking and vaping, duration of smoking and vaping, and several others (see supplementary file 1). 

#### 2.2.3. Product Characteristics Used

Product characteristics used were investigated by asking about e-liquid flavors used, nicotine concentrations, cigarette brands smoked and several others (see supplementary file 1). 

#### 2.2.4. Attractiveness and Reasons Related to Cigarettes and E-Cigarettes

Attractiveness and reasons related to cigarette and e-cigarette users were investigated by asking: ‘The E-cigarette is attractive because (more than one answer possible)’. Similar, reasons for cigarette use were investigated by asking: ‘Which of the following statements applies to you? I smoke/I used to smoke: (More than one answer possible)’ (see supplementary file 1).

#### 2.2.5. Socio-Cognitive Factors

To investigate socio-cognitive factors related to smoking, vaping, and not smoking and vaping, items were included in triplicate to investigate vaping, smoking behavior and not using e-cigarettes or cigarettes. For example, the attitude was assessed with four semantic-differentials about not smoking or vaping, cigarette smoking, and e-cigarette use by asking if participants thought ‘not smoking or vaping was good or bad on a 7-point Likert scale’. To establish scales for attitude, deliberation, trust in information provision, social ties, self-efficacy, and barriers of accessibility of e-cigarettes a reliability analysis was performed to establish scales. If the reliability analysis showed sufficient internal consistency (Cronbach’s alpha α ≥ 0.60), then items were included in a scale. An overview of internal consistency of scales can be found in supplementary file 1. Unless otherwise stated, all determinants used a 7-point Likert scale as answer options, such as either 1 equals totally disagree to 7 equals totally agree or semantic-differentials, such as 1 equals really bad to 7 equals really good.

### 2.3. Data Analysis

SPSS data were exported and further preprocessed in Microsoft Excel for statistical data analysis in R statistical software version 3.5.1 [42] using the random Forest package. Descriptive analyses were performed to gain insight into the participant characteristics of the study sample. Chi-square tests and analyses of variance (t-tests) were performed to assess differences in characteristics between dual users and exclusive e-cigarette users.

To determine which aspects were important to distinguish a dual user from an e-cigarette user, analyses were performed using random forest [43]. Random forest (RF) is a machine learning algorithm that classifies an outcome (dual use versus e-cigarette use) of an individual using an ensemble of decision trees with predictor variables (including demographics, smoking and vaping behavior, product characteristics, attractiveness and reasons related to cigarettes and e-cigarettes, and socio-cognitive factors). For the RF, a data table with 163 items and concepts (features) for 80 dual users and 36 exclusive e-cigarette users was used. Using 5-fold cross-validation, the data were randomly split into subsets containing approximately the same ratio of dual users and e-cigarette users: In a training set (80% of the data) and test set (20% of the data). In each cross-validation the training set was used to build an RF classifying model consisting of 1000 trees to predict if a participant in the test set is either a dual user or an exclusive e-cigarette user, this was done five times. After the five cross-validation runs, the overall prediction accuracy was calculated. Additionally, RF assessed the relative importance of each prediction variable by determining how much the error increased as a result of random rearrangement of the data for that variable (R settings: Type = 1, scale = TRUE). The resulting variable importance factor (averaged per factor across the five cross-validations) was used to calculate the corresponding *p*-value, which was adjusted to the Benjamini-Hochberg False Discovery Rate (FDR) [44] to correct for multiple testing. The variables with FDR adjusted *p*-values (FDR < 0.05) were used for a new round of RF classification to confirm the accuracy that could be obtained with these markers.

## 3. Results

### 3.1. Participants Characteristics

Of the 116 dual users and exclusive e-cigarette users, 43.1% was male and 56.9% female, 28.4% was highly educated (26.7% low education level, and 44.8% middle education level), and the average age was 49.1 (±12.5, min = 21, max = 79). Age did not significantly differ between dual users and exclusive e-cigarette users.

### 3.2. Differences between Dual Users and Exclusive E-Cigarette Users

All differences between dual users and exclusive e-cigarettes on the 163 predictor variables included in our analysis are reported in supplementary file 2. Table 1 reports the significant differences found between dual users and exclusive e-cigarette users. 

#### 3.2.1. Differences in Past and Current Smoking and Vaping Behavior

Significant differences in tobacco and e-cigarette behavior were observed (see Table 1) between dual users (that currently both smoke and vape) and exclusive e-cigarette users (former smokers that are currently only vaper-only) concerning onset of tobacco smoking, quantity of tobacco cigarettes smoked in their past or current smoking behavior, and lifetime status of tobacco smoking (Fagerstrom index) (*p* < 0.05). All exclusive e-cigarette users smoked more than 100 cigarettes in their lifetime. The onset of smoking was longer ago among e-cigarette users (i.e., more pack years) than dual users. In addition, when they still smoked (n = 36, all current e-cigarette users have a history of smoking), exclusive e-cigarette users smoked more cigarettes a day than current dual users smoke at the moment of the survey (*p* < 0.05). No differences were observed in how long dual users and exclusive e-cigarette users have been vaping (i.e., onset of vaping).

#### 3.2.2. Differences in Product Characteristics Used

No significant differences between dual users and exclusive e-cigarette users were observed in product characteristics used, such as current or first used concentrations of nicotine in e-liquids, or e-liquid flavors (see supplementary file 2). 

#### 3.2.3. Differences in Attractiveness and Reasons Related to Cigarettes and E-Cigarettes

Differences were observed between dual users and exclusive e-cigarette users in the perceived attractiveness of e-cigarettes (see Table 1). Compared to exclusive e-cigarette users, dual users more often found e-cigarettes to look nice, and the variety of e-liquid flavors appealing (*p* < 0.05). Dual users reported more often than exclusive e-cigarette users that avoiding smoking restrictions and the novelty of e-cigarettes were reasons to vape (*p* < 0.05). For cigarettes, dual users found the flavor of tobacco and the variety of brands to be more attractive than exclusive e-cigarette users (*p* < 0.05). E-cigarette users found the adjustable settings and nicotine concentrations of e-cigarette more attractive than dual users (*p* < 0.05). Compared to dual users, e-cigarette users reported health benefits and e-cigarettes as an alternative for smoking more often as reasons for e-cigarette use (*p* < 0.05). In addition, e-cigarette users found the cigarette to be unattractive more often than dual users (*p* < 0.05). In particular, they found it unattractive how smokers smell after smoking a cigarette (*p* < 0.05). No differences were observed in unattractive product characteristics of the e-cigarette, such as the design, the e-liquid flavors, or the price.

#### 3.2.4. Differences in Socio-Cognitive Factors 

E-cigarette users had a more positive attitude towards e-cigarettes and had a more negative attitude towards smoking compared to dual users (*p* < 0.05) (see Table 1). Compared to exclusive e-cigarette users, dual users felt more connected—their social ties with smokers were stronger—to other smokers and they more often had a partner, family, friends, and colleagues who smoked (Social influence (f), *p* < 0.05). Dual users had higher levels of deliberation about not smoking or vaping, and although they had a higher intention to quit vaping than exclusive e-cigarette users (*p* < 0.05), their self-efficacy to quit vaping and smoking was lower (*p* < 0.05). Dual users also perceive fewer risks related to smoking compared to e-cigarette users (*p* < 0.05). Significant differences were also found in information-seeking behavior, dual users less often used a Dutch Vape forum as a source of information than exclusive e-cigarette users (*p* < 0.05), and they would like to receive more information about e-liquids than exclusive e-cigarette users (*p* < 0.05). Dual users would find information independent if it was presented under the auspices of the national government (*p* < 0.05). Finally, exclusive e-cigarette users would find information more independent and reliable if researchers report no conflict of interest (*p* < 0.05).

### 3.3. Identifying Unique Factors that Discriminate Dual User from an Exclusive E-Cigarette User

**Figure 1:**
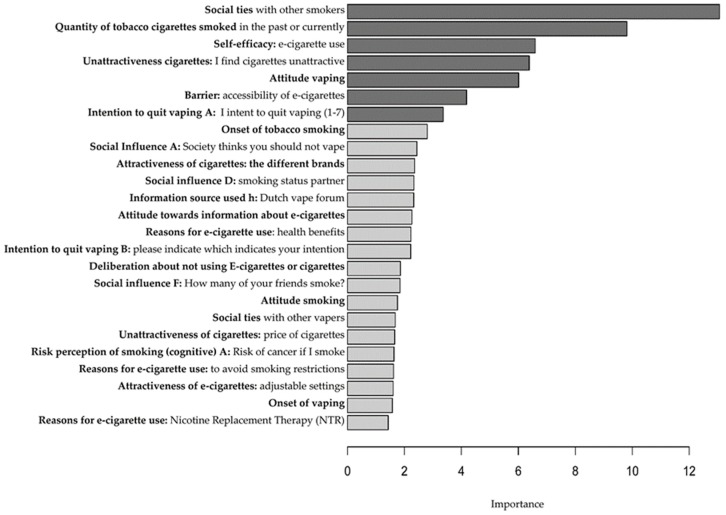
Top twenty-five factors to distinguish between dual users and e-cigarette users. Bar lengths indicate the variable importance factors, the seven significant (FDR 5%) factors are shown in dark gray.

Random forest analysis on dual use versus e-cigarette use identified 7 of the 163 factors as contributing significantly to the prediction accuracy (FDR 5%). Together, these seven factors allowed for 86.2% prediction accuracy. Figure 1 shows the 25 highest-ranking relevant distinguishing features, with the top seven significant factors after adjusting for multiple testing. The seven most relevant factors to distinguish a dual user from an exclusive e-cigarette user are (FDR 5%): Social ties with other smokers, quantity of tobacco cigarettes smoked in the past (e-cigarette users) or currently (dual users), self-efficacy to not vape and smoke, unattractiveness of cigarettes, attitude towards e-cigarettes, barriers: accessibility of e-cigarettes, and intention to quit vaping (A) (see Table 1 for significant differences between dual users and exclusive e-cigarette use).

## 4. Discussion

We used random forest—a machine learning algorithm—to identify important and unique distinguishing features between dual users and exclusive e-cigarette users. Based on the random forest, adult, current dual users and adult, current exclusive e-cigarette users can be distinguished from each other with 86.2% accuracy based on three out of four included clusters of factors: Current and past smoking behavior, unattractiveness of cigarette product characteristics, and socio-cognitive factors regarding smoking, vaping, and not smoking and vaping. Similar to previous research [9,23,24], our random forest analysis found no distinguishing features between dual users and exclusive e-cigarette users in product characteristics used.

First, regarding current and past smoking behavior, previous research found that dual users reduce the number of cigarettes smoked a day [25,45]. Our findings also indicate that adult, current dual users (n = 80) currently smoked fewer cigarettes a day than e-cigarette users did in the past, when they were cigarette smokers (n = 36) [25,45]. Research found that the number of cigarettes smoked per day increased the likelihood of initiating e-cigarette use [9,22], the reduced level of current smoking quantity a day among dual users may hint at a transitory phase of switching completely to exclusive e-cigarette use [46]. Similar to PATH [47] studies in the US, longitudinal research in the Netherlands is needed to investigate transitory phases of smoking and vaping to increase our understanding of dual use and exclusive e-cigarette use.

Second, exclusive e-cigarette users found cigarettes more unattractive than dual users. This unattractive aspect of cigarettes was a distinguishing feature between dual users and exclusive e-cigarette users. Further research is necessary to understand if increasing the unattractiveness of cigarettes might facilitate smokers—including dual users—to switch to exclusive e-cigarette use. Additionally, similarly to earlier results, dual users found the variety of e-liquid flavors available an attractive characteristic of both e-cigarettes and cigarettes, which is similar to earlier results, and they more often than exclusive e-cigarette users vape to avoid smoking restrictions [19,25,26,27,28,29]. Consequently, research is needed to gain insight into attractive aspects of e-cigarettes and unattractive aspects of cigarettes for smokers and dual users to identify factors that health communication can target. By targeting the identified distinguishing factors, health communication strategies can stress the pros of e-cigarettes and the cons of cigarettes for smokers and dual users. Furthermore, research into attractive and unattractive aspects of cigarettes and e-cigarettes is needed among never smokers and vapers to target preventive health communication strategies at vulnerable non-smokers and non-vapers [19,24,30].

Third, in contrast with previous research [23], our results show that various socio-cognitive factors are important to distinguish a dual user from an exclusive e-cigarette user. Dual users experienced more social ties with other smokers than exclusive e-cigarette users, and contrary to dual users, exclusive e-cigarette users had higher levels of perceived self-efficacy to not smoke or vape [30,34]. In addition, dual users had a more negative attitude towards e-cigarettes than exclusive e-cigarette users, which is supported by previous research [24]. Lastly, dual users, more often than exclusive e-cigarette users, had a partner who smoked. These factors could be targeted with health communication to aid smokers and dual users who want to switch to exclusive e-cigarette use or quit smoking.

Finally, the results from our random forest analysis provide insight into factors that had a unique contribution in distinguishing the dual users from exclusive e-cigarette users, which may have a practical implication for the improvement of health communication about smoking and vaping. Further research is needed to test effective communication strategies that target these factors to prevent the adverse health effects of dual use. The seven features provide a first insight into the most relevant features that health communication may be able to target to aid dual users who would like to switch to exclusive use in the future, or prevent dual use among exclusive users. Health communication strategies that stress the pros of e-cigarettes for cigarette smokers; that provide tools on how to cope with the pressure of a social environment to smoke cigarettes; and that provide tools on how to better cope with not smoking cigarettes to increase motivation to fully switch from cigarettes to e-cigarettes should be tested. Factors that can be targeted in health communication could focus on supporting smoking cessation among smokers and dual users by managing outcome expectancies of e-cigarette use compared to cigarette smoking, improving social ties of smokers and dual users with exclusive e-cigarette users [24,30,34], making cigarettes seem less attractive [48], strengthening positive attitudes towards e-cigarettes as a smoking cessation tool with inoculation messages and build resistance for a pro-smoking social environment of smokers and dual users [49], and providing guidance in the deliberation process of smoking cessation [50].

### 4.1. Limitations

To ensure one-time smokers or exclusive e-cigarette users were excluded in our analysis, the subset of dual users and exclusive e-cigarette users was relatively small, but sufficient in size for classification by methods, such as random forest. Due to our small sample, further research is needed to investigate the generalizability of the features found in the current study. In addition, due to the cross-sectional study design, no causal conclusions can be drawn on the most relevant distinguishing features. Selection bias may have occurred, as an online panel was used, which include individuals who are motivated to fill in questionnaires.

## 5. Conclusions

This study combined factors from four different research angles into a single machine learning analysis to identify the most relevant distinguishing features for discerning a dual user from an exclusive e-cigarette user. Our results demonstrate that it is a combination of features from three clusters that discern a dual user from an exclusive e-cigarette user: Current and past smoking behavior, the unattractiveness of cigarette product characteristics, and socio-cognitive factors regarding smoking and vaping. This particular combination provides new and relevant information regarding the differences between dual users and e-cigarettes users that can be applied to develop improved health communication targeted to prevent adverse health effects related to dual use.

## Figures and Tables

**Table 1:** Significant differences between dual users and exclusive e-cigarette users.

	Dual Users (n = 80)	E-Cigarette Users (n = 636)
(1) Past and current smoking and vaping behavior	n = 80	n = 36
Quantity of tobacco cigarettes smoked in the past (e-cigarette users) or currently (dual users)	Current smoking quantity ^A^	Past smoking quantity ^A^
*< ½ package p. day*	16.3%	2.8%
*½ −1 package p. day*	63.7%	36.1%
*1 package p. day*	13.8%	22.2%
*> 1 package p. day*	6.3%	38.9%
Lifetime status of tobacco smoking		
*<100 cigarettes in life*	6.30%	0.00%
*>100 cigarettes in life*	93.70%	100.00%
Onset of tobacco smoking		
*<6 months*	1.3%	2.8%
*6−12 months*	1.3%	0.0%
*1−5 years*	8.8%	0.0%
*5−10 years*	11.3%	0.0%
*>10 years*	77.5%	97.2%
Onset of vaping		
*<6 months*	35.0%	13.9%
*6−12 months*	20.0%	16.7%
*1−5 years*	41.3%	61.1%
*5−10 years*	3.8%	8.3%
		
(2) Product characteristics used	n = 80	
*No significant differences were found*		
(3) Attractiveness and reasons related to cigarettes and e-cigarettes	n = 80	n = 36
Attractiveness of e-cigarettes		
*The product looks nice*	18.8%	5.6%
*Due to all the different flavors*	46.3%	25.0%
*Because it is possible to alter the setting of the E-cigarette to my wishes*	20.0%	38.9%
*Because the nicotine level can be varied*	45.0%	66.7%
*Not applicable, I do not find the E-cigarette/vaper attractive*	8.8%	0%
Attractive characteristics of cigarettes (yes%)		
*The product looks nice*	7.5%	0%
*Due to all the different flavors*	30.0%	11.1%
*Because you can smoke different brands*	26.3%	5.6%
*Not applicable, I do not find the cigarette attractive*	28.8%	72.2%
Unattractiveness of cigarettes		
*The price of the product*	35.0%	58.3%
*Because you stink after you have smoked a cigarette*	43.8%	66.7%
Reasons for e-cigarette use		
*For their health advantages: effects on health, fewer ingredients than a cigarette.*	42.25%	72.2%
*As an alternative to cigarettes: it is like the smoking or because of the throat hit*	28.8%	50.0%
*To get round the smoking ban (to be able to vape in places where smoking is normally forbidden).*	22.5%	5.6%
*To try something new: out of curiosity about new products, different flavors, different apparatus/designs, for pleasure, as a hobby, or because it is cool/trendy/classy.*	11.3%	0%
(4) Socio-cognitive factors regarding cigarette use, e-cigarette use, and not smoking or vaping	n = 80	n = 36
Attitude towards e-cigarettes	4.5 (0.8)	5.0(0.8)
Attitude towards smoking	3.5(1.2)	2.7(1.2)
Barrier: accessibility of e-cigarettes	5.2(1.1)	4.8(1.5)
Deliberation not using e-cigarettes and cigarettes	4.5(1.4)	3.9(1.3)
Independency of information: (e)	50.0%	72.2%
Independency of information: (h)	18.8%	5.6%
Information need: (j)	7.9%	0%
Information source used: not applicable	6.3%	0%
Information source used (h)	6.3%	22.2%
Intention to quit vaping A.	3.8(2.0)	2.9(1.8)
Intention to quit vaping B.	3.6(2.4)	2.3(1.8)
Reliability of information (e)	51.3%	75.0%
Risk perception of smoking (cognitive) A	4.7(1.3)	5.3(1.1)
Self-efficacy to not vape or smoke	3.9(1.2)	4.8(1.2)
Social Influence A	4.2(1.7)	3.2(1.6)
Social influence D	33.8%	13.9%
Social influence F	3.6(1.1)	4.1(1.1)
Social Ties with other smokers (n = 79)	3.3(1.1)	2.3(1.2)

Note: Data on are presented as means (SD) or percentages of dual users, and exclusive e-cigarette users. All factors presented here are significant. A complete overview of all included variables in the random forest can be found in supplementary file 1. ^A^ Quantity of tobacco cigarettes smoked in the past or currently displays the current smoking behavior of dual users, and the past smoking behavior of exclusive e-cigarette users before they switched to exclusive e-cigarette use; Independency of information: (e) If the researchers themselves have no vested interest in the results; Independency of information: (h) If the research is under the auspices of the national government, if there is a governmental logo; Information need: (j) I would like more information about the e-liquids available; Information source used: Not applicable: I never search for information; Information source used: (h) Dutch Vape forum or Acvoda (Active for vaping); Intention to quit vaping A. Please indicate on a scale from 1 to 7 your intent to quit vaping in the next 6 months; Intention to quit vaping B. Please indicate which of the statements indicates your intention best; Reliability of information: (e) I find research reliable if the researchers have no conflict of interest to declare; Risk perception of smoking (cognitive) A: If I smoke, then my risk of developing some form of cancer during my lifetime is…; Social Influence A: Society thinks that you should not smoke E-cigarettes; Social influence D: Smoking status partner (% of yes); Social Influence F: How many of your family, friends or colleagues use cigarettes?

## Supplementary Materials

The following are available online at https://www.mdpi.com/1660-4601/16/21/4191/s1, Supplementary file 1: survey; Supplementary file 2: differences between dual users and exclusive e-cigarette users.
