# Supplementary material for: A Combination of Factors Related to Smoking Behavior, Attractive Product Characteristics, and Socio-Cognitive Factors are Important to Distinguish a Dual User from an Exclusive E-Cigarette User
Source: Int J Environ Res Public Health. 2019 Oct 30;16(21):4191. doi: 10.3390/ijerph16214191 (PMC6862614; doi:10.3390/ijerph16214191)
Supplement: Supplementary file 1 [file ijerph-16-04191-s001.zip › file 1 survey.pdf]

A Combination of Factors Related to Smoking Behavior, Attractive Product Characteristics, and Socio-Cognitive Factors are Important to Distinguish a Dual User from an Exclusive E-Cigarette User

Kim A.G.J. Romijnders [1,2,*], Jeroen L.A. Pennings [1], Liesbeth van Osch [2], Hein de Vries [2] and Reinskje Talhout [1]

[1] Centre for Health Protection, National Institute for Public Health and the Environment (RIVM), Antonie van Leeuwenhoeklaan 9, 3721 MA Bilthoven, The Netherlands; jeroen.pennings@rivm.nl (J.L.A.P.); Reinskje.talhout@rivm.nl (R.T.).

[2] Department of Health Promotion, CAPHRI School for Public Health and Primary Care, Maastricht University, POB 616 6200 MD Maastricht The Netherlands; Liesbeth.vanosch@maastrichtuniversity.nl (L.v.O); hein.devries@maastrichtuniversity.nl (H.d.V.).

* Correspondence: kim.romijnders@rivm.nl; Tel.: +31-30-274-4512

Appendix A

Table A.1 Overview of the included measures regarding demographics

Concept	Item	Answer option	Explanation of the concept	Reference
**Demographics**	How old are you?	Open question		Centraal Bureau voor Statistiek (CBS) [1]
	What is your gender?	Male-female		
	What is your level of education?	Did not finish school<br>• Primary school to 8th grade<br>• Some high school, did not graduate<br>• High school graduate, diploma or the equivalent (for example: GED)<br>• Some college credit, no degree<br>• Trade/technical/vocational training<br>• Associate degree<br>• Bachelor's degree<br>• Master's degree<br>• Professional degree<br>• Doctorate degree	Low level of education: answer options 1, 2, 3<br>Middle level of education: answer options 4, 5<br>Higher level of education: answer options 6, 7	

Table A.2 Smoking and vaping characteristics of participants

Concept	Item	Answer options	Explanation and formation of the concept	
**Type of user**	A. I smoke or vape	1. Both cigarettes and E-cigarettes<br>2. Only cigarettes<br>3. Only E-cigarettes<br>4. I do not smoke or vape<br>5. I have smoked in the past<br>6. I have vaped in the past<br>7. I have smoked both cigarettes and vaped in the past	• Response options A.4 formed the group non-users.<br>• Response options A.2 & B.1 OR B.2 formed smokers.<br>• Response options A.1 & (B.1 OR B.2) & C1.OR C2) formed dual users.<br>• Response options A.3 & Cs1 OR C.2 formed E-cigarette users.	Pearson, Hitchman, Brose, Bauld, Glasser, Villanti, McNeill, Abrams and Cohen [2] Amato, Boyle and Levy [3], International Tobacco Control Policy Evaluation Project [4]
	B. How often do you smoke?	1. Every day<br>2. Not every day, but at least once a week<br>3. Not every week, but at least once a month<br>4. Less than monthly		
	C. How often do you vape?	1. Every day<br>2. Not every day, but at least once a week<br>3. Not every week, but at least once a month<br>4. Less than monthly<br>5. I have never vaped regularly, I only tried it once or twice.		
**Smoking behavior**	How many cigarettes have you smoked in your life?	• Fewer than 100 cigarettes in my life<br>• 100 or more cigarettes in my life	Single item	Pearson, Hitchman,

	How long have you been smoking?	• 1 – 6 months<br>• Between 6 – 12 months<br>• Between 1 – 5 years<br>• More than 5 years	Single item	Brose, Bauld, Glasser, Villanti, McNeill, Abrams and Cohen [2], Amato, Boyle and Levy [3], International Tobacco Control Policy Evaluation Project [4], Heatherton, Kozlowski, Frecker and Fagerstrom [5]
	How often have you tried to quit smoking?	• Never<br>• 1 – 3 times<br>• More than 3 times<br>• Don't know	Single item	
	I smoked… (*number of cigarettes)*	• 5 – 10 (less than half a package)<br>• 10 – 13 (a bit more than half a package)<br>• 16 – 19 (about three quarters of a package)<br>• 1 package<br>• 19 – 25 (a bit more than a package)<br>• I never smoked regularly I only tried it once or twice.	Single item	
**Vaping behavior**	How long have you been vaping?	• 1 – 6 months<br>• Between 6 – 12 months<br>• Between 1 – 5 years<br>• More than 5 years	Single item	Pearson, Hitchman, Brose, Bauld, Glasser,

How often have you tried to quit vaping?	• Never<br>• 1 – 3 times<br>• More than 3 times<br>• Unknown		Villanti, McNeill, Abrams and Cohen [2] Amato, Boyle and Levy [3], International Tobacco Control Policy Evaluation Project [4]
I have vaped different flavors in different situations	1 = totally disagree to 7 = totally agree	Single item	
How much nicotine does your current e-liquid contain?	• I vape without nicotine<br>• 1- 8 mg/ml<br>• 9 – 14 mg/ml<br>• 15 – 24 mg/ml<br>• > 25 mg/ml<br>• > 36 mg/ml<br>• Don't know<br>• Not applicable	Single item	
How much nicotine did the first E-liquid you used contain?	• I vape without nicotine<br>• 1- 8 mg/ml<br>• 9 – 14 mg/ml<br>• 15 – 24 mg/ml<br>• > 25 mg/ml<br>• > 36 mg/ml<br>• Don't know<br>• Not applicable	Single item	
How likely is it that you will start using E-cigarettes /vapers in the future?	very likely --- very unlikely (1-7)	Single item	NA
If you have the opportunity, how likely is it that you will use E-cigarettes/vapers without nicotine in the future?		Single item	

	How many E-cigarettes/vapers are you currently using?	• Zero<br>• Two<br>• Three or more	Single item	
	How did you get to know about the E-cigarette?	• Via my family doctor/ practice nurse<br>• Internet<br>• Adverts on TV<br>• Via acquaintances/friends/family who use E-cigarettes.<br>• Advertisements	Single item	Romijnders, van Osch, de Vries and Talhout [6], Romijnders, van Osch, de Vries and Talhout [7]
	Sometimes you can have an unpleasant taste experience while vaping; this can be caused by vaping without E-fluid, for example. This is called a dry hit.			
	Do you or did you often have a problem with a dry hit?	• I don't know<br>• Yes, very often<br>• Yes, often Yes, sometimes<br>• Now and again<br>• No	Single item	Romijnders, van Osch, de Vries and Talhout [6], Romijnders, van Osch, de Vries and Talhout [7]
**Flavor preference of non-users and smokers**	If you were to start using an E-cigarette, which flavor would you like to try? *(check all that apply)*	• Tobacco.<br>• Menthol/mint<br>• Nuts<br>• Herbs, spices<br>• Coffee/tea	If participants selected 'I do not want to try a flavor', no other flavor could then be selected simultaneously.	Yingst, Veldheer, Hammett, Hrabovsky

	• Cocktails<br>• Alcohol, other<br>• Sodas<br>• Sweet, chocolate<br>• Sweet, vanilla<br>• Sweets, other<br>• Fruit<br>• Sweet, desserts<br>• Milk products<br>• Candy<br>• Floral<br>• Unflavored<br>• I do not want to try a flavor	The closed answer options that were used to assess flavor preference were recoded in accordance with the thirteen main categories of the recently published E-liquid flavor wheel [8], with the exception of "I don't want to try a flavor". Recoding reported flavor preferences resulted in the following thirteen main categories: tobacco (survey item: tobacco), menthol/mint (survey item: menthol/mint), nuts (survey item: nuts), spices (survey items: herbs, spices), coffee/tea (survey items: coffee; tea), alcohol (survey items: alcohol, cocktail; alcohol, other), other beverages (survey items: soda; sweet, other), fruit (survey item: fruit), dessert (survey items: sweet, dessert; milk product), other sweets (survey items: sweet, chocolate; sweet, vanilla),	and Foulds [9]

			candy (survey items: sweet, candy), other flavors (survey items: floral; other) and unflavored (survey item: unflavored). For example, if a non-user or smoker reported an interest in the survey items “sweet, candy” and “alcohol, cocktail”, their answers were recoded as a preference for the categories candy and alcohol, respectively. Open answers from dual and E-cigarette users were assessed for recoding of their closed answers in accordance with the categories of the e-liquid flavor wheel [8].	
**Smoking cessation information**	*By means of the following questions we would like to form an impression of the way you may potentially quit smoking. These questions only concern stopping smoking cigarettes.*			
	How many times have you tried to stop smoking?	• Never<br>• Once<br>• Twice<br>• Three times<br>• More than three times<br>• I don’t know	Single item	International Tobacco Control Policy Evaluation Project [4]

	Have you had anything to help you stop smoking? (more than one answer is possible)	• No, I haven't used an aid<br>• I have used the electronic cigarette as an aid<br>• Medicines<br>• Nicotine replacement therapy<br>• Self-help program<br>• Discussed stopping smoking with the family doctor<br>• Behavioral support programs<br>• Stop smoking course or group therapy<br>• Other therapies or aids<br>• Other aids or methods	Single item	
**Vaping cessation information**	*By means of the following questions, we would like to form an impression of the way you may potentially quit smoking. These questions only concern vapers/ E-cigarettes.*			
	How often have you tried to stop vaping?	• Never<br>• Once<br>• Twice<br>• Three times<br>• More than three times<br>• I don't know	Single item	International Tobacco Control Policy Evaluation Project [4]
	Choose the option or options that are applicable to you (more than one answer possible).			
	Since I have been vaping E-cigarettes cigarettes…	• … I am smoking fewer tobacco cigarettes<br>• … I smoke just as many cigarettes as I used to<br>• … I no longer smoke any cigarettes at all	Single item	International Tobacco Control Policy

		• … I smoke more/for longer in comparison with cigarettes<br>• … I feel physically more healthy<br>• … I have more of a craving to use nicotine<br>• … I feel mentally more healthy		Evaluation Project [4]
**Reasons for tobacco product use or e-cigarette use**	*Which of the following statements applies to you? I smoke/smoked/used e-cigarettes/vapers: (more than one answer possible)*	Because E-cigarettes/vapers are easy to get.	Single item	Romijnders, van Osch, de Vries and Talhout [6], Romijnders, van Osch, de Vries and Talhout [7]
		Because they are easy to use.		
		Due to the cost: less expensive than cigarettes/affordable		
		For their health advantages: effects on health, fewer ingredients than a cigarette.		
		As an aid to smoking fewer cigarettes or giving them up completely: a method of stopping smoking, to prevent me going back to cigarettes.		
		Because it is less addictive than cigarettes, variation in nicotine level is possible.		
		To cope with cravings and to prevent/cope with withdrawal symptoms		
		As an alternative to cigarettes: it is like the smoking or because of the throat hit (add info-button).		

	To get round the smoking ban (to be able to vape in places where smoking is normally forbidden).		
	To help me to focus and improve my performance, to reduce stress or to prevent weight gain.		
	Due to improved sense of taste and smell: because it tastes better than cigarettes, and to be discreet (you can hide the fact that you smoke, no unpleasant smells).		
	This is a real experience, one that can only be experienced by doing it.		
	To try something new: out of curiosity about new products, different flavors, different apparatus/designs, for pleasure, as a hobby, or because it is cool/trendy/classy.		
	For social reasons: because it was recommended by friends or family, due to pressure from the people around met, to extend my social network or because it is socially acceptable.		
Which of the following statements is applicable to you? I do not/no longer vape/use E-	Because it is addictive.	Single item	Romijnders, van Osch, de Vries and Talhout [6],
	Because of the disadvantages to health: Unsafe during pregnancy, injurious, injurious to other people, effects on health, another step towards		

cigarettes/vapers): (more than one answer possible)	smoking cigarettes, the ingredients in E-liquid, increase in weight.		Romijnders, van Osch, de Vries and Talhout [7]
	Because of the cost: costs too much or more expensive than cigarettes.		
	Due to practical failings: difficult to use, difficult to obtain, too different from smoking a cigarette, changing to other NRTs does not help to stop smoking or cravings, technical aspect (poor quality product), no throat hit.		
	Because it isn't cool		
	Because my friends don't do it either		
	Not interested, not curious		
Which of the following statements applies to you? I smoke/I used to smoke: (more than one answer possible)	Because cigarettes are easy to obtain.	Single item	Romijnders, van Osch, de Vries and Talhout [6], Romijnders, van Osch, de Vries and Talhout [7]
	Because they are easy to use.		
	To help me to focus and improve my performance, to reduce stress or to prevent weight gain.		
	This is a real experience, one that can only be experienced by doing it.		
	For social reasons: because it was recommended by friends and family, due to pressure from the people around me, to extend		

	my social network or because it is socially acceptable.		
	To have a moment for myself: a break from working or studying, just to do something completely different etc.		
	Because a cigarette tastes so good.		
	Due to stress: at work or at home, for example.		
	Because it gives me a good feeling.		
	Smoking is no more risky that a lot of other things that people do.		
	Due to the situation: After a meal, with a cup of coffee, with alcohol, on social occasions.		
Which of the following statements applies to you? I do not smoke / I no longer smoke: (more than one answer possible)	Because it damages the health of the people around me.	Single item	Romijnders, van Osch, de Vries and Talhout [6], Romijnders, van Osch, de Vries and Talhout [7]
	Because it is unhealthy.		
	Because it costs me a lot of money.		
	Because I am ashamed of smoking.		
	Because it takes a lot of my time.		
	Because it is addictive.		
	The product looks nice	Single item	

**Attractiveness of tobacco products and e-cigarettes**	*The E-cigarette/vaper is attractive because (more than one answer possible)*	Due to all the different flavors		Romijnders, van Osch, de Vries and Talhout [6], Romijnders, van Osch, de Vries and Talhout [7]
		Because it is possible to alter the setting of the E-cigarette to my wishes		
		Due to its varying designs		
		Due to the price of the product		
		Due to the price of the E-liquids		
		Because the nicotine level can be varied		
		Because you can blow nice smoke clouds with it		
		Not applicable, I do not find the E-cigarette/vaper attractive		
	The E-cigarette/vaper is unattractive because (more than one answer possible).	The appearance of the E-cigarette	Single item	Romijnders, van Osch, de Vries and Talhout [6], Romijnders, van Osch, de Vries and Talhout [7]
		Its many flavors		
		The price of the product		
		The price of the E-liquids		
		The weight of the E-cigarette (the apparatus)		
		The shape of the E-cigarette		
		How the product feels in my hand, it is different to a cigarette		

	Not applicable, I do not find the E-cigarette/vaper attractive		
I think the cigarette is attractive because… (more than one answer possible).	The product looks nice	Single item	Romijnders, van Osch, de Vries and Talhout [6], Romijnders, van Osch, de Vries and Talhout [7]
	Due to all the different flavors		
	Because you can smoke different brands		
	Due to the price of the product		
	Because smoking looks cool and classy		
	Because it looks cool when other people smoke		
	Because you can blow nice smoke clouds with it		
	Not applicable, I do not find the cigarette attractive		
The cigarette is unattractive because… (more than one answer possible).	The appearance of the cigarette	Single item	Romijnders, van Osch, de Vries and Talhout [6], Romijnders, van Osch, de Vries and Talhout [7]
	Its many flavors		
	The price of the product		
	The many brands		
	The smell of cigarettes		
	Packaging of cigarettes		

	Because you stink after you have smoked a cigarette	
	Not applicable, I find the cigarette attractive	

Table A.3 Socio-cognitive factors

Concept	Item	Answer option	Explanation of the scale	Formation of the concept	Cronbach's alpha (α)	Reference
**Knowledge about tobacco product use and e-cigarette use**	1. The E-cigarette/vaper is 95% less damaging than a cigarette.	0 = incorrect and 1 = correct<br><br>NA = don't know	0 = no correct answers to 9 = all statements were answered correctly	- 0 = no correct answers to 12 = all statements were answered correctly.<br>- The scores were summed to come to a final score of possible correct answers out of 12.<br>- *I don't know* was categorized as incorrect.<br>- The coding of correct and incorrect answers was based on scientific consensus.<br>- The statements assessing knowledge were based on previously conducted a focus group study.	NA	Romijnders, van Osch, de Vries and Talhout [7]
	2. Only water vapor comes out of an E-cigarette/vaper.					
	3. E-cigarettes/vapers are the same thing.					
	4. E-cigarette use can lead to irritation and damage of the airways, palpitations and an increased risk of cancer.					
	5. The E-cigarette/vaper is a scientifically proved means of stopping smoking.					
	6. There are just as many harmful substances in the E-cigarette liquid as in a cigarette.					
	7. Smoking increase the risk of developing various diseases, including lung cancer and various other types of cancer, cardiovascular disease and COPD.					
	8. Additives (substances added to the tobacco in a cigarette)					

	cannot make cigarette smoke any more addictive.					
	9. Smoking is the main cause of premature death.					
	10. It is always good to stop smoking, even for a short time.					
	11. Passive smoking is also damaging.					
	12. Getting enough exercise compensates for the risks to health from smoking.					
**Attitude towards E-cigarettes**	*I think vaping is…*		1= very negative towards E-cigarette use and 7 = very positive towards E-cigarette use.	- The four items were summed and averaged to compute one score of the concept attitude towards E-cigarettes.	*0.927*	Lehmann, de Melker, Timmermans and Mollema [11]
		Really bad (1) – Really good (7)				
		Really harmful (1) – Really safe (7)				
		Really gross (1) – Really nice (7)				
		Really socially unacceptable behavior (1)				

		– Really socially acceptable behavior (7)				
**Attitude towards smoking**	*I think smoking*		1= very negative towards smoking and 7 = very positive towards smoking.	- The four items were summed and averaged to compute one score of the concept attitude towards smoking.	0.889	Lehmann, de Melker, Timmermans and Mollema [11]<br>Montano and Kasprzyk [12]
		Really bad (1) – Really good (7)				
		Really harmful (1) – Really safe (7)				
		Really gross (1) – Really nice (7)				
		Really socially unacceptable behavior (1) – Really socially acceptable behavior (7)				
	I think not using E-cigarettes or cigarettes is…				0.940	

**Attitude towards not using E-cigarettes and cigarettes**	Really bad (1) – Really good (7)<br>Really harmful (1) – Really safe (7)<br>Really gross (1) – Really nice (7)<br>Really socially unacceptable behavior (1) – Really socially acceptable behavior (7)	1= very negative towards not using E-cigarettes and cigarettes and 7 = very positive towards not using E-cigarettes and cigarettes.	- The four items were added together and averaged to compute one score of the concept attitude towards not using E-cigarettes and cigarettes.		Lehmann, de Melker, Timmermans and Mollema [11]
**Deliberation about the pros and cons of tobacco product use**		1 = no deliberation about product use and 7 = deliberation	The nine items were summed and averaged to compute one score of the concept **Deliberation of the pros and cons of tobacco product use**	0.864	Lehmann, de Melker, Timmermans and Mollema [11]

			about product use			
**Deliberation on the pros and cons of E-cigarette use**	*Please keep your own decision in mind, but have you considered the option of using E-cigarettes?*		1 = no deliberation about E-cigarette use to 7 = very extensive deliberation about E-cigarette use	- The three items were summed and averaged to compute one score of the concept Deliberation on the pros and cons of e-cigarette use.	*0.* 656	Lehmann, de Melker, Timmermans and Mollema [11]
	I have visualized how it would feel not to smoke and not to vape.	1 = I have not visualized how I would feel 7 = I have visualized how I would feel				
	I have visualized how it would feel to smoke.	1 = I have not considered the consequences 7= I have considered the consequences				
	I have visualized how it would feel to vape.	1 = no conscious list of the pros and cons 7 = a very conscious list of pros and cons				
**Deliberation of the pros and cons of smoking.**	*Please keep your own decision in mind, but have you considered smoking?*		1 = no deliberation of the about smoking to 7 = very	- The three items were summed and averaged to compute one score of the concept Deliberation of the pros and cons of smoking	0.579	Lehmann, de Melker, Timmermans
	1. I have tried to consider the consequences of	1 = I have not visualized how I would feel to 7 = I				

	not smoking or vaping.	have visualized how I would feel	extensive deliberation about smoking.			and Mollema [11]
	2. I have tried to visualize the consequences of smoking.	1 = I have not considered the consequences to 7= I have considered the consequences				
	3. I have tried to visualize the consequences of vaping.	1 = no conscious list of the pros and cons to 7 = a very conscious list of pros and cons				
**Deliberation about not using E-cigarettes or cigarettes**	*Please keep your own decision in mind, but have you considered the option of not using E-cigarettes and cigarettes?*		1 = no deliberation about not using E-cigarettes or cigarettes to 7 = very extensive deliberation about not using E-cigarettes or cigarettes.	- The three items were summed and averaged to compute one score of the concept Deliberation on not using e-cigarettes and cigarettes.	0.666	Lehmann, de Melker, Timmermans and Mollema [11]
	I have made/thought about a list of the pros and cons of not smoking or vaping.	1 = I have not visualized how I would feel about not using tobacco product and e-cigarettes 7 = I have visualized how I would feel				
	I have made/thought about a list of the pros and cons of not smoking.	1 = I have not considered the consequences 7= I have considered the consequences				

	I have made a list of the pros and cons of not vaping.	1 = no conscious list of the pros and cons 7 = a very conscious list of pros and cons				

Concept	Item	Answer option	Explanation of the scale	Formation of the concept	Cronbach's alpha (α)	Reference
**Risk perception about e-cigarette use (cognitive)**	A. If I vape, then my risk of developing some form of cancer during my lifetime is…	very small -- very big (1--7)	1 = low to 7 = perception of cognitive risk of susceptibility to health risks related to e-cigarette use	Answer options were recoded to make sure 7 is positive and 1 is negative.	NA	Janssen [7] de Vries, van Osch, Eijmael, Smerecnik and Candel [6]
	B. I think that if I vape, my risk of developing some form of cancer during my lifetime:			Cognitive susceptibility and severity items on side-effects could not be summed [6] because Cronbach's alpha was below .6. A concept could not be created so the individual item was used.		
**Risk perception of e-cigarette use (affective)**	A. My feeling is that if I vape, the risk of developing some form of cancer during my lifetime:	very small -- very big (1--7)	1 = low to 7 = affective risk perception for susceptibility to health risks related	Answer options were recoded to make sure 7 is positive and 1 is negative.	NA	Janssen [7] de Vries, van Osch, Eijmael, Smerecnik and Candel [6]

			to E-cigarette use.			
**Risk perception of smoking (cognitive)**	A. If I smoke, then my risk of developing some form of cancer during my lifetime is…<br>B. I think that if I smoke, my risk of developing some form of cancer during my lifetime:	very small -- very big (1--7)	1 = low to 7 = cognitive risk perception for susceptibility to health risks related to smoking.	Answer options were recoded to make sure 7 is positive and 1 is negative.<br>Cognitive susceptibility and severity items on side-effects could not be summed [6] because Cronbach's alpha was below .6. A concept could not be created so the individual item was used.	NA	Janssen [7] de Vries, van Osch, Eijmael, Smerecnik and Candel [6]
**Risk perception of smoking (affective)**	A. A. My feeling is that if I smoke, the risk of developing some form of cancer during my lifetime:	very small -- very big (1--7)	1 = low to 7 = affective risk perception for susceptibility to health risks related to smoking.	Answer options were recoded to make sure 7 is positive and 1 is negative.	NA	Janssen [7] de Vries, van Osch, Eijmael, Smerecnik and Candel [6]
**Risk perception of not using E-cigarettes or**	A. How big a risk do you think you have of developing some form of cancer	very small -- very big (1--7)	1 = low to 7 = cognitive risk perception for susceptibility	Answer options were recoded to make sure 7 is positive and 1 is negative.	NA	Janssen [7] de Vries, van Osch, Eijmael, Smerecnik

**cigarettes (cognitive)**	during your lifetime?<br>B. If I don't smoke or vape, then the risk that I will develop some form of cancer during my life time is…<br>C. I think that my risk of developing some form of cancer during my lifetime:		to health risks related to no use.	Cognitive susceptibility and severity items on side-effects could not be summed [6] because Cronbach's alpha was below .6. A concept could not be created so the individual item was used.		and Candel [6]
**Risk perception of not using E-cigarettes or cigarettes (affective)**	A. My feeling is that the risk of developing some form of cancer during my lifetime:<br>B. My feeling is that if don't smoke or vape, then the risk of developing some	very small -- very big (1--7)	1 = low to 7 = affective risk perception for susceptibility to health risks related to no use.	Answer options were recoded to make sure 7 is positive and 1 is negative.<br>Affective susceptibility and severity items on side-effects could not be summed [6] because Cronbach's alpha was below .6. A concept could not be	NA	Janssen [7] de Vries, van Osch, Eijmael, Smerecnik and Candel [6]

	form of cancer during my lifetime ….		created so the individual item was used.		
**Information seeking behavior**	**Independency of information** *I think that scientific research into E-cigarettes/vapers, is independent … (more than one answer possible).*	Check all that apply	Frequencies were used to determine what participants thought about independent scientific research. The most-reported response option was used as the definition of independent.	NA	Romijnders, van Osch, de Vries and Talhout [7]
	Only if researchers have influence on how the research is carried out.				
	Only if researchers have influence on how the research results are interpreted.				
	Only if researchers have influence on how the research results are communicated.				
	If all the research results are made known and not just the desired results.				
	If the researchers themselves have no vested interests in the results.				
	If the research is not financed by the tobacco industry.				
	If the research is under the auspices of the national government, if there is a governmental logo.				

**Information seeking behavior**	**Reliability of information** *I think that scientific research into E-cigarettes/vapers is reliable … (more than one answer possible).*	Check all that apply	Frequencies were used to determine what participants thought about independent scientific research.	NA	
	Only if researchers have influence on how the research is carried out		The most-reported response option was used as the definition of independent.		
	Only if researchers have influence on how the research results are interpreted				
	Only if researchers have influence on how the research results are communicated				
	If all the research results are made known and not just the desired results				
	If the research is not financed by the tobacco industry				
	If the research is under the auspices of the national government, if there is a governmental logo				
**Information seeking behavior**	**Information source** *I think the most important source of information about the E-cigarette/vapers is:*	*Maximum of 3 answers possible*	Frequencies were used to determine which sources were used.	NA	Romijnders, van Osch, de Vries and Talhout [7]<br><br>Sandefer, Westra, Khairat,

		Pieczkiewicz and Speedie [10]
	Television	
	Newspaper	
	Radio	
	Advertizing (signs, shop displays, advertizing folders, pop-ups, YouTube advertisments, advertizing banners)	
	Internet	
	Dutch National Institute for Public Health and the Environment (RIVM)	
	Facebook or Twitter	
	Dutch Vape forum or Acvoda (*Active for vaping*)	
	Friends, family, acquaintances or colleagues	
	Health care professionals, such as my family doctor or practice nurse	
	Not applicable. I never look for information about the E-cigarette/vapers	

**Information seeking behavior**	**Information need** *I would like to receive more information about:…*	Check all that apply	Frequencies were used to determine what participants wanted to know about e-cigarettes.	NA	Romijnders, van Osch, de Vries and Talhout [7]
	a: The harmfulness of e-cigarettes				
	b: Where you can by e-cigarettes				
	c: E-liquids				
	d: The different types of e-cigarettes available				
	e: How an e-cigarette works				
	f: Whether the e-cigarette is an effective smoking cessation tool				
	g: How much e-liquid do you use on average with an e-cigarette				
	h: What settings to use with an e-cigarette				
	i: How often on a day you should use e-cigarettes				
	j: About the e-liquids available				
	All the above				

	I do not want additional information about the e-cigarette					
**Attitude towards information about e-cigarettes**	In my opinion, the information about the E-cigarette/vaper is:		Very negative indeed – very positive indeed (1-7)	Single item		
	In my opinion, the information about the E-cigarette/vaper is:		Not at all useful - very useful indeed (1--7)			
**Trust**	I think RIVM is trustworthy.	completely agree -- completely disagree (1-7)	1 low to 7 = high level of trust in information provided by the Dutch National Institute of Public Health and the	The two items were summed and averaged to compute one score of the concept **Trust.**	0.915	Siegrist, Earle and Gutscher [13]
	I think RIVM is independent.					

			Environment.			
**Social Ties: e-cigarette users**	*How often does it happen that…?*		1 low to 7 high level of social ties with other vapers		0.900	Thoits [14]
	A vaper greets me even though I don't know him/her?	Not at all often (1) – very often (7)				
	People try to point out the advantages of vaping?					
	Non-vapers come to stand with you when you are using an E-cigarette/vaping?					
	People ask you what your vaper is?					
	People respond negatively to vaping?					
	To what extent do you agree with the following comments?					
	I feel a bond with E-cigarette users.	completely agree -- completely disagree				

	I feel I am an E-cigarette user.			
**Social Ties: smokers**	*How often does it happen that…?*		0.868	Thoits [14]
	A smoker greets me even though I don’t know him/her?	Not at all often – very often		
	Non-smokers come to stand with you when you are smoking?			
	People ask you what brand of cigarette you are smoking?			
	People respond negatively to smoking?			
	To what extent do you agree with the following comments?			
	I feel a bond with smokers.	completely agree -- completely disagree		
	I feel I am a smoker.			

**Social influence**	A: Society thinks that you should not smoke E-cigarettes.	completely agree (1) – (7) completely disagree	Single item	NA	Montano and Kasprzyk [12]
	B: Society thinks that you should not smoke.	completely agree (1) – (7) completely disagree			
	C: My partner uses E-cigarettes/vapers.	Yes (1), No (0)			
	D: My partner smokes.	Yes (1), No (0)			
	E: How many of your family, friends or colleagues use E-cigarettes/vapers?	• (almost) All of them<br>• More than half<br>• Half<br>• Less than half<br>• (almost+) No-one<br>• Not applicable			
	F: How many of your family, friends or colleagues use cigarettes?	• (almost) All of them<br>• More than half<br>• Half<br>• Less than half<br>• (almost+) No-one<br>• Not applicable			

**Self-efficacy: e-cigarette use**	I am sure that I will not start to use E-cigarettes or vape.	1 = very low perceived self-efficacy to 7 = very high perceived self-effcacy to quit smoking and vaping	Items were summed and averaged in one concept for self-efficacy: e-cigarette use	0.868	Dijkstra and Vries [15]
	If I vaped, then I am sure that I would be able not to vape (E-cigarette or vaper) and smoke				
**Self-efficacy: smoking**	I am sure that I will not start smoking.			0.631	Dijkstra and Vries [15]
	If I smoked, I am sure that I would be able not to vape or smoke E-cigarettes.				
	If I smoked, I am sure that I wouldbe				

	able to smoke E-cigarettes/vape.					
**Self-efficacy: not using tobacco products or e-cigarettes**	I am sure that I will not start to use E-cigarettes or vape, or start smoking.				0.847	Dijkstra and Vries [15]
	If I didn't smoke or vape (E-cigarette/vaper) then I am sure that I would be able not vape.					
**Barrier: accessibility of e-cigarettes**	E-cigarettes are easy to obtain.	1 = totally disagree to 7 = totally agree	1 = low perception of accessablity to e-cigaretts to 7 = high perception of accessibility to e-cigarettes	The tree items were summed and averaged to create a final score for the scale A**ccessibility**.	0.882	Romijnders, van Osch, de Vries and Talhout [7]
	E-liquids are easy to obtain.					
	Vapors are easy to obtain.*	* in the Netherlands a specific type of e-cigarette is marketed as a separate product without nicotine and in many fruit flavors. This device is very				

		similar in looks to the first generation e-cigarette and is not refillable. This specific type of e-cigarette is very popular among non-using adolescents.				
**Intention to quit**	A. Please indicate on a scale from 1 to 7 your intent to quit vaping in the next 6 months.	(1) I do not intent to quit vaping to (7) I intent to quit vaping.	1 = low intention to quit vaping to 7 = high intention to quit vaping	Single item	NA	Montano and Kasprzyk [12]
	B. Please indicate which of the statements indicates your intention best	(1) I do not intent to quit vaping to (7) I really intent to quit vaping	1 = low intention to quit vaping to 7 = high intention to quit vaping	Single item	NA	Montano and Kasprzyk [12]

References

[1] Centraal Bureau voor Statistiek (CBS), International Standard Classification of Education (ISCED) [Dutch version], Standaard onderwijsindeling, Centraal Bureau voor Statistiek (CBS), Heerlen, 2018, p. 39,

[2] J.L. Pearson, S.C. Hitchman, L.S. Brose, L. Bauld, A.M. Glasser, A.C. Villanti, A. McNeill, D.B. Abrams, J.E. Cohen, Recommended core items to assess e-cigarette use in population-based surveys, Tob Control 27(3) (2018) 341-346.

[3] M.S. Amato, R.G. Boyle, D. Levy, How to define e-cigarette prevalence? Finding clues in the use frequency distribution, Tob Control 25(e1) (2016) e24-9.

[4] International Tobacco Control Policy Evaluation Project, ITC Netherlands (NL11_Pw), 2017. https://www.itcproject.org/files/ITC_NL11_Replenishment_S_web_Eng_Dut.pdf. (Accessed 10, Jan, 2018 2018).

[5] T.F. Heatherton, L.T. Kozlowski, R.C. Frecker, K.O. Fagerstrom, The Fagerstrom Test for Nicotine Dependence: a revision of the Fagerstrom Tolerance Questionnaire, British journal of addiction 86(9) (1991) 1119-27.

[6] K. Romijnders, L. van Osch, H. de Vries, R. Talhout, Perceptions and Reasons Regarding E-Cigarette Use among Users and Non-Users: A Narrative Literature Review, Int J Environ Res Public Health 15(6) (2018).

[7] K. Romijnders, L. van Osch, H. de Vries, R. Talhout, A Deliberate Choice? Exploring the Decision to Switch from Cigarettes to E-Cigarettes, Int J Environ Res Public Health 16(4) (2019).

[8] E.J.Z. Krusemann, S. Boesveldt, K. de Graaf, R. Talhout, An E-liquid Flavor Wheel: A Shared Vocabulary based on Systematically Reviewing E-liquid Flavor Classifications in Literature, Nicotine Tob Res (2018).

[9] J.M. Yingst, S. Veldheer, E. Hammett, S. Hrabovsky, J. Foulds, A Method for Classifying User-Reported Electronic Cigarette Liquid Flavors, Nicotine Tob Res 19(11) (2017) 1381-1385.

[10] R.H. Sandefer, B.L. Westra, S.S. Khairat, D.S. Pieczkiewicz, S.M. Speedie, Determinants of Consumer eHealth Information Seeking Behavior, AMIA Annu Symp Proc 2015 (2015) 1121-9.

[11] B.A. Lehmann, H.E. de Melker, D.R.M. Timmermans, L. Mollema, Informed decision making in the context of childhood immunization, Patient Educ Couns 100(12) (2017) 2339-2345.

[12] D.E. Montano, D. Kasprzyk, Theory of Reasoned Action, Theory of Planned Behavior, and the Integrated Behavioral Model, in: K. Glanz, B.K. Rimer, K. Viswanath (Eds.), Health Behavior and Health Education: Theory, Research and Practice, John Wiley & Sons, New Jersey, 2008, pp. 67-96.

[13] M. Siegrist, T.C. Earle, H. Gutscher, Test of a trust and confidence model in the applied context of electromagnetic field (EMF) risks, Risk Anal 23(4) (2003) 705-16.

[14] P.A. Thoits, Mechanisms linking social ties and support to physical and mental health, J Health Soc Behav 52(2) (2011) 145-61.

[15] A. Dijkstra, H.D. Vries, Self-efficacy expectations with regard to different tasks in smoking cessation, Psychology & Health 15(4) (2000) 501-511.
