# Supplementary material for: A Combination of Factors Related to Smoking Behavior, Attractive Product Characteristics, and Socio-Cognitive Factors are Important to Distinguish a Dual User from an Exclusive E-Cigarette User
Source: Int J Environ Res Public Health. 2019 Oct 30;16(21):4191. doi: 10.3390/ijerph16214191 (PMC6862614; doi:10.3390/ijerph16214191)
Supplement: Supplementary file 1 [file ijerph-16-04191-s001.zip › file 2 differences.pdf]

A Combination of Factors Related to Smoking Behavior, Attractive Product Characteristics, and Socio-Cognitive Factors are Important to Distinguish a Dual User from an Exclusive E-Cigarette User

Kim A.G.J. Romijnders [1,2,*], Jeroen L.A. Pennings [1], Liesbeth van Osch [2], Hein de Vries [2] and Reinskje Talhout [1]

1 Centre for Health Protection, National Institute for Public Health and the Environment (RIVM), Antonie van Leeuwenhoeklaan 9, 3721 MA Bilthoven, The Netherlands; jeroen.pennings@rivm.nl (J.L.A.P.); Reinskje.talhout@rivm.nl (R.T.).
2 Department of Health Promotion, CAPHRI School for Public Health and Primary Care, Maastricht University, POB 616 6200 MD Maastricht The Netherlands; Liesbeth.vanosch@maastrichtuniversity.nl (L.v.O); hein.devries@maastrichtuniversity.nl (H.d.V.).
*** Correspondence: kim.romijnders@rivm.nl; Tel.: +31-30-274-4512**

Supplementary table 1 differences between dual users and exclusive e-cigarette users

Item	Dual users (n)	Dual users (mean/percentage)	Dual users (SD)	E-cigarette users (n)	E-cigarette users (mean/percentage)	E-cigarette users (SD)	p-value
**3.1. Participant Characteristics**							
Age	80	47.8	13.40	36	51.9	9.80	0.10
Gender	80			36			0.31
male		46.3%			36.1%		
female		53.8%			63.9%		
Education	80			36			0.70
low		30.0%			19.4%		
middle		40.0%			55.6%		
high		30.0%			25.0%		
**3.2.1. Differences in past and current smoking and vaping behavior**							
lifetime status of tobacco smoking (Fagerstrom index)	80			36			0.02
< 100 cigarettes in life		6.30%			0.00%		
> 100 cigarettes in life		93.70%			100.00%		
onset of tobacco smoking	80			36			0.06
< 6 months		1.3%			2.8%		
6-12 months		1.3%			0.0%		
1-5 years		8.8%			0.0%		
5-10 years		11.3%			0.0%		
> 10 years		77.5%			97.2%		
quantity of tobacco cigarettes smoked in their past (e-cigarette users) or current (dual users) smoking behavior	80			36			0.00
< ½ package/day		16.3%			2.8%		
½-1 package/day		63.7%			36.1%		
1 package/day		13.8%			22.2%		
> 1 package/day		6.3%			38.9%		
onset of vaping	80			36			0.01
< 6 months		35.0%			13.9%		
6-12 months		20.0%			16.7%		
1-5 years		41.3%			61.1%		
5-10 years		3.8%			8.3%		
vaping status ml	80	18.35	18.99	36	16.78	18.92	0.68
vaping status liquid	80			36			0.75
less than half a container		67.5%			77.8%		
a little bit more than half a container		15.0%			2.8%		
about three quarters of a container		7.5%			11.1%		
a little bit less than a container		2.5%			2.8%		
a container at max		2.5%			0.0%		
little more than one container		1.3%			0.0%		
**3.2.2. Differences in product characteristics used**							
Flavors situation	80	3.6	2.08	36	3.14	2.45	0.29
nicotine current				36			0.96

None		17.5%			8.30%		
1-8 mg/mL		27.5%			41.70%		
9-14 mg/mL		18.8%			16.70%		
15-24 mg/mL		25.0%			25.00%		
> 25 mg/mL		2.5%			0		
> 36 mg/mL		1.3%			2.80%		
nicotine first	80			36			0.48
None		13.8%			8.3%		
1-8 mg/mL		17.5%			13.9%		
9-14 mg/mL		25.0%			25.0%		
15-24 mg/mL		26.3%			41.7%		
> 25 mg/mL		3.8%			2.8%		
> 36 mg/mL		1.3%			1.6%		
future intention e-cigarette use	80	4.75	1.89	36	5.25	1.95	0.20
future intention e-cigarette nicotine content	80	4.24	1.94	36	4.33	1.80	0.80
quantity e-cigarettes used	80			36			0.32
I don't own my own e-cigarette		2.5%			5.6%		
1		68.8%			52.8%		
2		22.5%			30.6%		
more than 3		6.3%			11.1%		
type e-cigarette used	80			36			0.80
Disposable e-cigarettes		58.8%			58.3%		
Refillable and chargeable e-cigarettes		18.8%			13.9%		
Mods		8.8%			13.9%		
other		13.8%			13.9%		
AWARENESS FIRST	80			36			0.88
GP		0.0%			2.8%		
Internet		32.5%			30.6%		
Commercials on tv		7.5%			0.0%		
Through my social network		47.5%			55.6%		
Advertisements		6.3%			2.8%		
Other		6.3%			8.3%		
DRYHIT	80			61			0.13
very often		2.5%			5.6%		
often		8.8%			16.7%		
sometimes		17.5%			25.0%		
rarely		20.0%			47.2%		
never		40.0%			36.1%		
unknown		11.3%			5.6%		
**Current e-liquid flavors used (% of flavor category used)**							
FLAVOR TOBACCO	79	55.7%		35	54.3%		0.89
FLAVOR MENTHOL	79	24.1%		35	11.4%		0.09
FLAVOR NUTS	79	0.0%		35	0.0%		NA
FLAVOR SPICES	79	0.0%		35	0.0%		NA
FLAVOR COFFEE or TEA	79	3.8%		35	2.9%		0.79
FLAVOR ALCOHOL	79	0.0%		35	0.0%		NA
FLAVOR OTHER BEVERAGES	79	0.0%		35	0.0%		NA
FLAVOR FRUIT	79	8.9%		35	11.4%		0.69
FLAVOR DESSERT	79	1.3%		35	2.9%		0.61
FLAVOR CANDY	79	0.0%		35	5.7%		0.16
FLAVOR OTHER SWEETS	79	6.3%		35	5.7%		0.90
FLAVOR UNFLAVORED	79	0.0%		35	2.9%		0.32
FLAVOR OTHER FLAVOR	79	0.0%		35	2.9%		0.32
**First e-liquid flavors used (% of flavor category used)**							
FLAVOR TOBACCO	79	62.0%		35	74.3%		0.19
FLAVOR MENTHOL	79	19.0%		35	17.1%		0.81
FLAVOR NUTS	79	0.0%		35	0.0%		NA
FLAVOR SPICES	79	0.0%		35	0.0%		NA
FLAVOR COFFEE or TEA	79	2.5%		35	2.9%		0.92
FLAVOR ALCOHOL	79	1.3%		35	0.0%		0.32
FLAVOR OTHER BEVERAGES	79	0.0%		35	0.0%		NA
FLAVOR FRUIT	79	6.3%		35	2.9%		0.38
FLAVOR DESSERT	79	0.0%		35	0.0%		NA
FLAVOR CANDY	79	1.3%		35	0.0%		0.32
FLAVOR OTHER SWEETS	79	7.6%		35	0.0%		0.01
FLAVOR OTHER FLAVOR	79	0.0%		35	2.9%		0.32
FLAVOR UNFLAVORED	79	0.0%		35	0.0%		NA
FLAVOR UNFLAVORED	79	0.0%		35	0.0%		NA

3.2.3. Differences in attractiveness and reasons related to cigarettes and e-cigarettes

**Attractiveness of e-cigarettes**					
The product looks nice	80	18.8%	36	5.6%	0.03
Due to all the different flavors	80	46.3%	36	25.0%	0.02
Because it is possible to alter the setting of the E-cigarette to my wishes	80	20.0%	36	38.9%	0.05
Due to its varying designs	80	10.0%	36	2.8%	0.10
Due to the price of the product	80	22.5%	36	13.9%	0.25
Due to the price of the E-liquids	80	28.8%	36	36.1%	0.45
Because the nicotine level can be varied	80	45.0%	36	66.7%	0.03
Because you can blow nice smoke clouds with it	80	2.5%	36	0.0%	0.16
Not applicable, I do not find the E-cigarette/vaper attractive	80	8.8%	36	0.0%	0.01
**Unattractiveness of e-cigarettes**					
The appearance of the E-cigarette	80	17.5%	36	22.2%	0.57
Its many flavors	80	15.0%	36	11.1%	0.56
The price of the product	80	22.5%	36	13.9%	0.25
The price of the E-liquids	80	15.0%	36	16.7%	0.82
The weight of the E-cigarette (the apparatus)	80	17.5%	36	8.3%	0.15
The shape of the E-cigarette	80	8.8%	36	11.1%	0.70
How the product feels in my hand, it is different to a cigarette	80	20.0%	36	19.4%	0.95
Not applicable, I do not find the E-cigarette/vaper attractive	80	27.5%	36	36.1%	0.37
**Attractiveness of cigarettes**					
The product looks nice	80	7.5%	36	0.0%	0.01
Due to all the different flavors	80	30.0%	36	11.1%	0.01
Because you can smoke different brands	80	26.3%	36	5.6%	0.00
Due to the price of the product	80	7.5%	36	2.8%	0.25
Because smoking looks cool and classy	80	6.3%	36	2.8%	0.37
Because it looks cool when other people smoke	80	3.8%	36	0.0%	0.08
Because you can blow nice smoke clouds with it	80	1.3%	36	2.8%	0.62
Not applicable, I do not find the cigarette attractive	80	28.8%	36	72.2%	0.00
**Unattractiveness of cigarettes**					
The appearance of the cigarette	80	5.0%	36	2.8%	0.55
Its many flavors	80	8.8%	36	11.1%	0.70
The price of the product	80	35.0%	36	58.3%	0.02
The many brands	80	8.8%	36	2.8%	0.16
The smell of cigarettes	80	31.3%	36	38.9%	0.44
Packaging of cigarettes	80	10.0%	36	5.6%	0.39
Because you stink after you have smoked a cigarette	80	43.8%	36	66.7%	0.02
Not applicable, I find the cigarette attractive	80	13.8%	36	5.6%	0.14
**Reasons for e-cigarette use**					
Because E-cigarettes/vapers are easy to get.	80.00	5.0%	36.00	11.1%	0.30
Because they are easy to use.	80.00	27.5%	36.00	19.4%	0.34
Due to the cost: less expensive than cigarettes/affordable	80.00	41.3%	36.00	52.8%	0.26
For their health advantages: effects on health, fewer ingredients than a cigarette.	80.00	42.5%	36.00	72.2%	0.00
As an aid to smoking fewer cigarettes or giving them up completely: a method of stopping smoking, to prevent me going back to cigarettes.	80.00	42.5%	36.00	61.1%	0.07
Because it is less addictive than cigarettes, variation in nicotine level is possible.	80.00	28.8%	36.00	36.1%	0.45
To cope with cravings and to prevent/cope with withdrawal symptoms	80.00	11.3%	36.00	13.9%	0.70
As an alternative to cigarettes: it is like the smoking or because of the throat hit (add info-button).	80.00	28.8%	36.00	50.0%	0.04
To get round the smoking ban (to be able to vape in places where smoking is normally forbidden).	80.00	22.5%	36.00	5.6%	0.01
To help me to focus and improve my performance, to reduce stress or to prevent weight gain.	80.00	7.5%	36.00	5.6%	0.69

Due to improved sense of taste and smell: because it tastes better than cigarettes, and to be discreet (you can hide the fact that you smoke, no unpleasant smells).	80.00	13.8%		36.00	22.2%		0.30
This is a real experience, one that can only be experienced by doing it.	80.00	1.3%		36.00	5.6%		0.30
To try something new: out of curiosity about new products, different flavors, different apparatus/designs, for pleasure, as a hobby, or because it is cool/trendy/classy.	80.00	11.3%		36.00	0.0%		0.00
For social reasons: because it was recommended by friends or family, due to pressure from the people around met, to extend my social network or because it is socially acceptable.	80.00	10.0%		36.00	5.6%		0.39
NA	80.00	2.5%		36.00	0.0%		0.16

3.2.4. Differences in socio-cognitive factors

Socio-cognitive factors

Attitude never use	80	5.15	1.23	36	5.34	1.14	0.43
Attitude smoking	80	3.28	1.11	36	2.69	1.17	0.01
Attitude vaping	80	4.50	0.75	36	5.03	0.82	0.00
Barrier: accessibility of e-cigarettes	80	5.17	1.13	36	4.84	1.52	0.25
Deliberation about not using E-cigarettes or cigarettes	80	4.48	1.42	36	3.92	1.28	0.04
Deliberation about the pros and cons of tobacco product use	80	4.43	1.24	36	4.02	1.29	0.11
Deliberation of the pros and cons of smoking.	80	4.50	1.30	36	4.07	1.56	0.16
Deliberation on the pros and cons of E-cigarette use	80	4.32	1.31	36	4.07	1.33	0.37
Intention to quit vaping A. Please indicate on a scale from 1 to 7 your intent to quit vaping in the next 6 months.	80	3.76	2.00	36	2.86	1.82	0.02
Intention to quit vaping B. Please indicate which of the statements indicates your intention best	80	3.56	2.38	36	2.25	1.81	0.00
Knowledge	80	8.54	1.92	36	8.61	1.40	0.82
Risk perception about e-cigarette use (cognitive): A. If I vape, then my risk of developing some form of cancer during my lifetime is…	80	4.29	1.17	36	4.25	1.02	0.86
Risk perception about e-cigarette use (cognitive): B. I think that if I vape, my risk of developing some form of cancer during my lifetime:	80	4.30	1.32	36	4.28	1.34	0.93
Risk perception of e-cigarette use (affective): A. My feeling is that if I vape, the risk of developing some form of cancer during my lifetime:	80	4.34	1.30	36	4.22	0.99	0.60
Risk perception of not using E-cigarettes or cigarettes (affective)A. My feeling is that the risk of developing some form of cancer during my lifetime:	80	4.44	1.29	36	4.36	1.13	0.75
Risk perception of not using E-cigarettes or cigarettes (affective): A. My feeling is that if don't smoke or vape, then the risk of developing some form of cancer during my lifetime ….	80	4.13	1.19	36	4.19	1.28	0.78
Risk perception of not using E-cigarettes or cigarettes (cognitive): A. How big a risk do you think you have of developing some form of cancer during your lifetime?	80	4.54	1.26	36	4.83	1.21	0.23
Risk perception of **not** using E-cigarettes or cigarettes (cognitive): B. If I don't smoke or vape, then the risk that I will develop some form of cancer during my life time is…	80	3.93	1.24	36	4.08	1.11	0.49
Risk perception of not using E-cigarettes or cigarettes (cognitive): C. I think that my risk of developing some form of cancer during my lifetime:	80	4.29	1.32	36	4.36	1.05	0.75
Risk perception of smoking (cognitive): A. If I smoke, then my risk of developing some form of cancer during my lifetime is…	80	5.08	1.29	36	5.22	1.31	0.58
Risk perception smoking (affective): My feeling is that if I smoke, the risk of developing some form of cancer during my lifetime:	80	4.71	1.34	36	5.11	1.30	0.14
Risk perception smoking (cognitive): B. I think that if I smoke, my risk of developing some form of cancer during my lifetime:	80	4.73	1.28	36	5.31	1.06	0.01
Self-efficacy: e-cigarette use	80	3.89	1.21	36	4.84	1.02	0.00
Self-efficacy: not using tobacco products or e-cigarettes	80	3.37	1.34	36	3.77	1.26	0.12
Self-efficacy: smoking	80	3.88	1.18	36	3.57	1.37	0.23
Social Influence A: Society thinks that you should not vape E-cigarettes.	80	4.21	1.68	36	3.22	1.57	0.00

Social Influence B: Society thinks that you should not smoke.	80	5.56	1.52	36	5.31	2.05	0.50
Social Influence C: Vaping status partner (% of yes): My partner uses E-cigarettes/vapers.	80			36			0.14
Social influence D: smoking status partner (% of yes): My partner smokes.	80	33.8%		36	13.9%		0.01
social influence E: How many of your family, friends or colleagues use E-cigarettes/vapers?	80	4.41	0.98	36	4.61	0.93	0.30
social influence F: How many of your family, friends or colleagues use cigarettes?	80	3.59	1.13	36	4.06	1.12	0.04
SOCIAL TIES with other smokers	79	3.34	1.09	36	2.32	1.22	0.00
SOCIAL TIES with other vapers	80	3.00	1.24	36	3.09	0.99	0.68
TRUST in the National Institute of Public Health and the Environment	73	4.95	1.25	34	4.43	1.61	0.10
**Information seeking behavior**							
Source of information used a: Television	80.00	13.8%		36.00	8.3%		0.37
Source of information used b: Newspaper	80.00	8.8%		36.00	8.3%		0.94
Source of information used c: Radio	80.00	1.3%		36.00	2.8%		0.62
Source of information used d: Advertising (signs, shop displays, advertising folders, pop-ups, YouTube advertisements, advertising banners)	80.00	6.3%		36.00	2.8%		0.37
Source of information used e: Internet	80.00	67.5%		36.00	80.6%		0.13
Source of information used f: Dutch National Institute for Public Health and the Environment (RIVM)	80.00	5.0%		36.00	2.8%		0.55
Source of information used g: Facebook or Twitter	80.00	1.3%		36.00	0.0%		0.32
Source of information used h: Dutch Vape forum or Acvoda (Active for vaping)	80.00	6.3%		36.00	22.2%		0.04
Source of information used i: Friends, family, acquaintances or colleagues	80.00	30.0%		36.00	33.3%		0.73
Source of information used j: Health care professionals, such as my family doctor or practice nurse	80.00	5.0%		36.00	11.1%		0.30
Source of information used k: Not applicable. I never look for information about the E-cigarette/vapers	80.00	6.3%		36.00	0.0%		0.02
Need of information a: The harmfulness of e-cigarettes	76.00	38.2%		36.00	47.2%		0.37
Need of information b: Where you can by e-cigarettes	76.00	11.8%		36.00	19.4%		0.33
Need of information c: E-liquids	76.00	13.2%		36.00	8.3%		0.43
Need of information d: The different types of e-cigarettes available	76.00	9.2%		36.00	11.1%		0.76
Need of information e: How an e-cigarette works	76.00	10.5%		36.00	8.3%		0.71
Need of information f: Whether the e-cigarette is an effective smoking cessation tool	76.00	21.1%		36.00	27.8%		0.45
Need of information g: How much e-liquid do you use on average with an e-cigarette	76.00	9.2%		36.00	8.3%		0.88
Need of information h: What settings to use with an e-cigarette	76.00	3.9%		36.00	0.0%		0.08
Need of information i: How often on a day you should use e-cigarettes	76.00	6.6%		36.00	2.8%		0.34
Need of information j: About the e-liquids available	76.00	7.9%		36.00	0.0%		0.01
Need of information all: All the above	76.00	15.8%		36.00	8.3%		0.24
Need of information none: I do not want additional information about the e-cigarette	76.00	21.1%		36.00	19.4%		0.84
Attitude towards information about e-cigarettes	76	4.54	1.11	36	4.42	1.39	0.63
Reliability of information a: Only if researchers have influence on how the research is carried out	80	32.5%		36	27.8%		0.61
Reliability of information b: Only if researchers have influence on how the research results are interpreted	80	26.3%		36	22.2%		0.64
Reliability of information c: Only if researchers have influence on how the research results are communicated	80	27.5%		36	19.4%		0.34
Reliability of information d: If all the research results are made known and not just the desired results	80	66.3%		36	75.0%		0.34
Reliability of information e: If the researchers have no conflict of interest to declare	80	51.3%		36	75.0%		0.01
Reliability of information f: If the research is not financed by the tobacco industry	80	37.5%		36	55.6%		0.08
Reliability of information g: If the research is under the auspices of the national government, if there is a governmental logo	80	16.3%		36	8.3%		0.21
Independence of information a: Only if researchers have influence on how the research is carried out.	80	35.0%		36	36.1%		0.91
Independence of information b: Only if researchers have influence on how the research results are interpreted.	80	27.5%		36	27.8%		0.98

Independence of information c: Only if researchers have influence on how the research results are communicated.	80	27.5%	36	22.2%	0.54
Independence of information d: If all the research results are made known and not just the desired results.	80	62.5%	36	72.2%	0.30
Independence of information e: If the researchers themselves have no vested interests in the results.	80	50.0%	36	72.2%	0.02
Independence of information f: If the research is not financed by the tobacco industry.	80	38.8%	36	50.0%	0.27
Independence of information h: If the research is under the auspices of the national government, if there is a governmental logo.	80	18.8%	36	5.6%	0.03
